# Caffeine and Exercise: What Next?

**DOI:** 10.1007/s40279-019-01101-0

**Published:** 2019-04-11

**Authors:** Craig Pickering, Jozo Grgic

**Affiliations:** 1 Institute of Coaching and Performance, School of Sport and Wellbeing, University of Central Lancashire, Fylde Road, Preston, PR1 2HE UK; 2 The Prenetics DNAFit Research Centre, London, UK; 3 Institute for Health and Sport (IHES), Victoria University, Melbourne, Australia

## Abstract

Caffeine is a widely utilized performance-enhancing supplement used by athletes and non-athletes alike. In recent years, a number of meta-analyses have demonstrated that caffeine’s ergogenic effects on exercise performance are well-established and well-replicated, appearing consistent across a broad range of exercise modalities. As such, it is clear that caffeine is an ergogenic aid—but can we further explore the context of this ergogenic aid in order to better inform practice? We propose that future research should aim to better understand the nuances of caffeine use within sport and exercise. Here, we propose a number of areas for exploration within future caffeine research. These include an understanding of the effects of training status, habitual caffeine use, time of day, age, and sex on caffeine ergogenicity, as well as further insight into the modifying effects of genotype. We also propose that a better understanding of the wider, non-direct effects of caffeine on exercise, such as how it modifies sleep, anxiety, and post-exercise recovery, will ensure athletes can maximize the performance benefits of caffeine supplementation during both training and competition. Whilst not exhaustive, we hope that the questions provided within this manuscript will prompt researchers to explore areas with the potential to have a large impact on caffeine use in the future.

## Key Points

**Table:** 

Caffeine is a well-replicated performance-enhancing supplement, with these effects established at meta-analysis level; as such, further research exploring the straightforward ergogenic effects of caffeine is unlikely to alter practice.
However, there are many unanswered questions with regard to the use of caffeine in sport which represent promising avenues to enhance our understanding and provide some nuance into the use of caffeine around exercise.
These unanswered questions include whether the ergogenic effects of caffeine alter with sex, time of day, genotype, habitual use, and training status, and there is a need for a greater understanding of the effects of caffeine on performance anxiety and post-exercise recovery.

## Introduction

Caffeine (1,3,7-trimethylxanthine) is a popular ergogenic aid, widely used by athletes at all levels [1, 2]. The performance-enhancing effects of caffeine have been studied for over 100 years, with the first known study on the subject published in 1907 [3]. Since these early studies, interest in caffeine has developed, to the point that it is now one of the most well-established ergogenic aids, with performance-enhancing effects across a wide range of exercise modalities [4].

Several meta-analyses examining the effects of caffeine ingestion on exercise performance have been conducted, exploring the effects of caffeine on a broad array of exercise tests, including 1 repetition maximum (1 RM) strength [5, 6], isokinetic peak torque [7], vertical jump height [6], power output across different exercise types [8–10], aerobic endurance performance [8, 11–15], and muscular endurance [5, 16]. The statistically significant effect sizes from these individual meta-analyses ranged from 0.16 (for isokinetic peak torque [7]) to 0.51 (for aerobic exercise performance [13]), suggesting that caffeine can reliably enhance performance. Further systematic reviews have highlighted an ergogenic effect of caffeine on sport-specific endurance [17], power-based sports [18], and resistance exercise [19, 20]. Two meta-analyses [21, 22] have reported no effect of caffeine on sprint and repeated sprint performance, although a number of individual studies utilizing either caffeine-containing energy drinks [23] or caffeine alone [24–27] have demonstrated a potentially ergogenic effect of caffeine on both, suggesting a need for further research in this area.

Alongside its well-established effects on a variety of physical performance tasks, caffeine also exerts acute cognitive benefits upon ingestion, especially in sleep-deprived subjects. This has been explored in military personnel, with caffeine demonstrated to improve cognitive aspects such as vigilance, memory, and mood, along with physical performance, both during overnight operations and following sleep restriction [28–30], results which have been replicated in the general public (for review, see Ruxton [31]). Similar findings have been reported in athletes. For example, Cook and colleagues [32] reported that caffeine doses of 1 and 5 mg/kg ameliorated loss of skill performance in elite rugby players following sleep restriction. In non-sleep restricted subjects, caffeine may enhance sports-specific skill performance (for review, see Baker et al. [33]). Furthermore, athletes are prone to mental fatigue, which can impair physical [34, 35] and sport-specific skill-based performance [36] and cognitive ability [37]. In mentally fatigued individuals, caffeine has been shown to enhance endurance performance [38] along with skill performance and cognitive function in sport-specific situations [39, 40], although this latter finding is equivocal [41, 42].

Given caffeine’s popularity, both in its use by athletes and its interest to researchers, it is tempting to believe that we potentially know all there is to understand about the use of caffeine in sport. The purpose of this review is to explore some areas where our knowledge of caffeine’s performance benefits is not clear, prompting potential directions for researchers to explore in the future.

## What Else Do We Need to Know About Caffeine in Sport?

At present, the performance-enhancing effects of caffeine on a plethora of exercise modes are well-established at meta-analysis level [5–16]. As such, it is clear that caffeine is an effective ergogenic aid—but can we better understand the context around its use to better inform practice? We propose that future research should instead attempt to explore the nuances of caffeine use. Given the high prevalence of caffeine ingestion amongst athletes [1, 2], such an approach is likely to yield additional performance enhancement. General caffeine guidelines recommend the consumption of 3–6 mg/kg of caffeine, typically 60 min before the start of exercise [43]; however, recently it has become apparent that there is considerable inter-individual variation in response to such a standardized protocol, with a variety of factors potentially driving this variation [44]. A greater understanding of these factors, explored in the following sections, will, hopefully, allow for the enhanced personalization of caffeine usage guidelines in the future.

### What are the Wider, Non-direct Influences of Caffeine on Performance?

Historically, the interest in caffeine within sports science has been on its performance-enhancing effects. However, it is important to understand the wider contexts of caffeine use, and the potential impact these could have on performance. For example, caffeine has been shown to potentially elevate feelings of anxiety [45], which is an important consideration for athletes—does pre-competition caffeine consumption increase anxiety to the extent that it becomes performance limiting? An individualized approach may be required here; some individuals may need an increase in arousal prior to some competitions, and caffeine may be an effective way to achieve this [46]. Conversely, for major competitions where anxiety and arousal are likely to be increased, pre-competition caffeine may need to be reduced, or avoided altogether, in order to protect performance [44].

Caffeine ingestion can also impact the subsequent ability to both fall asleep and achieve high-quality sleep [47, 48]. This effect is potentially prolonged, with Drake and colleagues [47] reporting that 400 mg of caffeine, ingested 6 h prior to bedtime, disrupted sleep quality, reduced sleep duration, and increased sleep latency. This is of interest within sporting contexts, where competitions often occur in the evening; here, pre-competition caffeine use may have a carryover effect, reducing sleep quality and duration, and subsequently harming recovery. This was explored by Dunican and colleagues [48] in a Super Rugby team. Here, players consumed caffeine prior to an evening match; post-match salivary concentrations of caffeine were associated with an increase in sleep latency and a decrease in both sleep duration and efficiency that evening compared to baseline data. Further research is required to understand what effect, if any, caffeine-induced sleep loss may have on subsequent performance (for example, across a 2-day competition), as well as replicating these initial findings. Enhancing our understanding in this area will undoubtedly assist in the provision of individualized caffeine guidelines around competition, with a pragmatic approach most likely required; is the decrease in sleep quality following caffeine ingestion a worthwhile price to pay for enhanced performance?

Aside from affecting sleep, caffeine may also modify post-training and competition recovery. In a recent review, Loureiro and colleagues [49] reported conflicting data regarding the effects of caffeine on muscle glycogen recovery, suggesting a need for further research in this area. Interestingly, cafestol and caffeic acid—ingredients of coffee—appear to enhance muscle glycogen recovery [49], suggesting that the use of coffee as a means to receive caffeine pre-exercise may confer some additional benefits compared to other caffeinated mediums. In another review [20], the authors concluded that caffeine ingestion prior to resistance training may reduce the occurrence of delayed onset of muscle soreness, recommending further research to explore this. Similar results have been reported following endurance exercise [50]. However, caffeine appears to delay autonomic recovery following exercise [51, 52]. As such, future research should seek to better understand the effect of pre-exercise caffeine ingestion on post-exercise recovery, particularly given that pre-exercise caffeine intake may increase physical exertion and hence muscle damage and training load. Furthermore, a better understanding of the role of caffeine on muscle glycogen recovery may lead to the use of caffeine *post*-exercise as a method to enhance recovery, although the demonstrated negative effect on autonomic function would require consideration.

Furthermore, genetic variation (see Sect. 2.2) is likely to contribute to the effect of caffeine on these aspects, with single-nucleotide polymorphisms (SNPs) in *ADORA2A* and *DRD2* associated with caffeine-induced anxiety and sleep disturbances [53, 54], demonstrating an interaction between some of these remaining questions regarding the use of caffeine within sport.

### What are the Effects of Genotype on Caffeine Ergogenicity?

Three recent reviews have explored the potential impact of genetic variation on the ergogenic effects of caffeine on performance [44, 54, 55], with two SNPs in *CYP1A2* and *ADORA2A* emerging as potential candidates. Of these, *CYP1A2* is most well-explored, with at least nine studies examining its effect on caffeine ergogenicity [56–64]; for a detailed summary of these studies, readers are directed to Fulton et al. [54]. The majority of these studies utilized small sample sizes, potentially hampering their statistical power and the derivation of firm conclusions. This notion is mirrored within the results, with some studies reporting no effect of the polymorphism [52, 56, 57, 59, 61] and others a modifying effect, but in different directions [58, 60, 63, 64]. In the study with the largest cohort (*n* = 101), Guest and colleagues [58] reported that moderate (4 mg/kg) doses of caffeine were ergogenic for AA genotypes, ineffective for AC genotypes, and ergolytic for CC genotypes. *CYP1A2* encodes for cytochrome P450 1A2, the enzyme responsible for ~ 95% of all caffeine metabolism [65]. Subjects with the AA genotype tend to produce more of this enzyme, and hence metabolize caffeine quicker than AC and CC genotypes [66]. A potential proposed mechanism for the impact of *CYP1A2* on caffeine ergogenicity is that the downstream metabolites of caffeine (paraxanthine, theobromine, and theophylline) have additional ergogenic effects, which is why the fast-metabolizing AA genotypes experience a further advantage [63]. Additionally, as caffeine is a vasoconstrictor, CC genotypes might experience prolonged vasoconstriction, harming endurance performance [58]. If these mechanisms are correct—and further work should aim to elucidate this—then there is the potential that ingestion of caffeine a greater period of time prior to exercise could improve caffeine’s ergogenic effects in C allele carriers [67].

As research in this field evolves, we should be able to gain a broader understanding as to the genetic influence on the effects of caffeine, be that specifically from a performance standpoint [46] or wider aspects influencing performance, such as anxiety [68, 69], sleep disturbances [70], and habitual use [71]. There is the potential that SNPs within genes located within the dopaminergic (such as *DRD2* and *COMT* [69, 72]), adenosine (*AMPD1* [73]), and adrenergic (*ADRA1A*, *ADRA2B*, *ADRB1*, *ADRB2*, and *ADRB3* [73]) systems may contribute to the demonstrated inter-individual variation in response to an acute caffeine dose within sporting contexts. This aspect also has important implications for caffeine research; if genotype does modify caffeine’s ergogenic effects between individuals, then these individual differences, when averaged across groups, may be masked, providing misleading results. However, the addition of genetic information to caffeine-based research may be practically problematic, as it is costly, and we do not fully understand which genetic variants modify caffeine’s ergogenic effects, meaning any potential stratification based on genotype may be incomplete.

### Does Time of Day Impact the Ergogenic Effects of Caffeine?

There is a potential effect of circadian rhythm on performance, with some studies demonstrating that performance in a given task is better in the afternoon compared to the early morning [74–76]. Specifically, muscular abilities, such as strength, appear to peak in the evening hours [77]. For example, Guette et al. [78] reported significantly lower maximal torque production at 06:00 and 10:00 h (~ 90% of maximum values) compared to strength performance at 18:00 h (~ 99% of maximal values). As athletes often have to undertake training sessions, or even compete, early in the morning, there is an increased interest in strategies to off-set this morning performance decrement.

Given its stimulatory role, caffeine represents a potential method of mitigating the performance decrement seen in the morning hours [79], particularly as studies have demonstrated that caffeine can serve to preserve performance during periods of sleep deprivation [32, 80]. This hypothesis has been tested by a number of recent studies [79, 81–85], detailed in Table 1. Mora-Rodríguez and colleagues [81] demonstrated that morning caffeine ingestion increased performance to that of an evening resistance exercise session. In a later study, the same research group observed that caffeine enhanced performance in the morning but not in the evening hours [82], with similar results observed by Souissi et al. [84]. Interestingly, Mora-Rodríguez and colleagues [82] reported a higher incidence of side effects upon p.m. caffeine ingestion.

**Table 1:** Summary of studies that explored the effects of caffeine supplementation on exercise performance at different times of day

Reference	Sample	Chronotype assessment	Testing times of day	Caffeine dose	Performance metric	Main findings
Boyett et al.^a^ [83]	20 young men (also categorized as trained *n* = 7, and untrained *n *= 7)	Not performed	One caffeine and one placebo condition in the morning hours (between 06:00 and 10:00 h); one caffeine and one placebo condition in the evening hours (between 16:00 and 20:00 h)	6 mg/kg	Isokinetic knee extension and 3-km cycling time trial	Isokinetic peak torque at angular velocity of 30 °/s was ‘possibly’ enhanced when ingesting caffeine in the evening as compared to evening placebo ingestion; for cycling time trial, ingesting caffeine in the morning ‘very likely’ enhanced performance as compared to morning placebo ingestion; ingesting caffeine in the evening ‘possibly’ enhanced performance as compared to evening placebo ingestion; ingesting caffeine in the morning ‘likely’ enhanced performance more than ingesting caffeine in the evening; when analyzed based on training status, ingesting caffeine in the morning ‘likely’ enhanced performance as compared to morning ingestion of placebo in trained individuals; the evening caffeine vs placebo comparison produced ‘unclear’ effects; ingesting caffeine in the morning and evening hours ‘likely’ enhanced performance as compared to placebo; in untrained individuals, ingesting caffeine in the morning or evening ‘likely’ improved performance as compared to morning and evening ingestion of placebo
Lopes-Silva et al. [79]	13 physically active young men	None of the participants belonged to any extreme type as determined by the Horne and Ösberg self- questionnaire	One caffeine and one placebo condition in the morning hours (between 08:00 h); one caffeine and one placebo condition in the evening hours (18:00 h)	5 mg/kg	10 × 6 s cycle sprints	No differences in total work between caffeine and placebo conditions
Miller et al. [202]	188 young male students	The study included morning, evening, and intermediate types as determined by the Horne and Ösberg self- questionnaire	Randomized to testing performed at 08:00, 11:00, 14:00, 17:00, 20:00, or 23:00 h	1 and 3 mg/kg	Forearm flexor MVC	Increase in MVC strength occurred only in the morning hours and with 3 mg/kg of caffeine
Mora-Rodríguez et al. [81]	12 young resistance-trained men	Not performed	One caffeine and one placebo condition in the morning hours (10:00 h); one placebo condition in the evening hours (18:00 h)	3 mg/kg	Squat and bench press with loads that elicited barbell displacement of 1.00 m/s and with loads amounting to 75% of 1 RM; knee extension and hand MVC; knee extension electrically evoked MVC	In the squat exercise when using loads that elicited barbell displacement of 1.00 m/s, ingesting caffeine in the morning and placebo in the evening enhanced barbell velocity as compared to morning ingestion of placebo; for the bench press, ingesting placebo in the evening enhanced barbell velocity as compared to morning ingestion of placebo and caffeine; in both the squat and bench press exercises with loads of 75% 1 RM, ingesting caffeine in the morning or placebo in the evening enhanced barbell velocity as compared to morning ingestion of placebo; for knee extension and hand MVC, no differences were observed between the conditions; ingesting caffeine in the morning enhanced electrically evoked knee extension MVC as compared to morning ingestion of placebo
Mora-Rodríguez et al. [82]	13 young resistance-trained men	Not performed	One caffeine and one placebo condition in the morning hours (08:00 h); one caffeine and one placebo condition in the evening hours (18:00 h)	6 mg/kg	Squat and bench press with loads of 25, 50, 75, and 90% of 1 RM	In the squat exercise, ingesting placebo in the evening, caffeine in the morning, and caffeine in the evening hours enhanced barbell velocity with loads of 25, 50, and 75% of 1 RM as compared to ingesting placebo in the morning; no significant effects were observed in the squat exercise with 90% 1 RM and in the bench press exercise with any of the employed loads
Pataky et al.^a^ [60]	25 young men and 13 young women	Not performed	15 participants performed the placebo and caffeine testing sessions at 10:00 h or earlier, while 23 participants performed the placebo and caffeine testing sessions at time later than 10:00 h	6 mg/kg and/or mouth rinsing with 25 ml of caffeine solution	Power output during a 3-km cycling time trial	In the group performing the testing session before 10:00 h, ingestion of caffeine, mouth rinsing with caffeine, and ingestion of caffeine plus mouth rinsing with caffeine ‘very likely,’ ‘likely’ and ‘most likely’ improved power output; mouth rinsing with caffeine ‘possibly’ had an ergolytic effect on power output in those exercising at times later than 10:00 h; placebo ingestion plus mouth rinsing with placebo and mouth rinsing with placebo ‘very likely’ had a greater effect in those exercising at 10:00 h or earlier; caffeine ingestion plus mouth rinsing with caffeine ‘likely’ improved greater in the participants exercising at 10:00 h or earlier as compared to those exercising at times later than 10:00 h
Souissi et al. [84]	12 young Judo athletes	None of the participants belonged to any extreme type as determined by the Horne and Ösberg self- questionnaire	One caffeine and one placebo condition in the morning hours (07:00 h); one caffeine and one placebo condition in the evening hours (17:00 h)	5 mg/kg	30-s lower-body Wingate test	Peak and mean power were increased only when caffeine was ingested in the morning

*1* *RM* 1 repetition maximum, *MVC* maximal voluntary contraction

^a^Data were analyzed using the magnitude-based inferences approach

In another study [83], caffeine appeared to exert a greater ergogenic effect (+ 2.3%) on 3-km cycling time trial performance when consumed in the morning compared to the evening (+ 1.4%). Again, cycling performance is generally impaired in the morning versus evening hours [86], and these differences may explain the possible greater effects of caffeine ingestion in the morning. Caution must be exercised when interpreting these results, however, as the authors utilized the magnitude-based inference method which has recently received criticism due to inflated type I error rates [87]. Finally, two studies [79, 85] have reported no effect on time of day on caffeine ergogenicity.

The morning-evening differential in performance, and the potential modifying effect of caffeine ingestion, is an important methodological consideration for researchers to keep in mind, and report in future studies. A recent meta-analysis [7] reported an ergogenic effect of caffeine on isokinetic peak force (pooled effect size 0.16). Closer scrutiny of the methods used in the included studies indicates that only a single study [88] detailed the time of day at which the exercise testing was performed, which for both the caffeine and placebo trials was between 09:00 and 12:00 h. Of the ten included studies within that meta-analysis, this study reported the largest effect size (0.53) of caffeine on isokinetic strength, potentially suggesting that the time of day of caffeine ingestion represents an important variable influencing the magnitude of caffeine’s ergogenic effects.

Given the relatively sparse research around this question, future work should aim to better understand what role—if any—time of day has on the effects of caffeine. This information could further be utilized to enhance caffeine strategies for athletes, especially given the potential effects of caffeine on sleep disturbances (Sect. 2.1). This could also be important from the standpoint of travel across time zones, an increasingly frequent occurrence for high level athletes, and the associated jet lag, which has been shown to harm performance [89, 90]. Caffeine has been demonstrated to mitigate the negative effects of jet lag on performance [91] and daytime sleepiness [92], and may assist in the retraining of circadian rhythm following a large time change [89–94], although it remains understudied in this regard [95, 96]. Future studies carried out within the area of caffeine and circadian rhythms should consider exploring the impact of caffeine on jet lag management and time-shift in athletes. Furthermore, it would likely be worthwhile to assess whether an individual’s chronotype further modifies caffeine’s ergogenic effects across different times of day. Finally, researchers should report the timing of performance tests utilized within caffeine trials, in order to control for this potentially modifying variable.

### Does Caffeine Ergogenicity Vary According to Training Status?

It has been long perpetuated in the scientific literature that the effects of caffeine on exercise performance differ between trained and untrained individuals, popularized by the work by Collomp et al. [24]. Here, seven trained and seven recreational swimmers had their swimming performance tested on two occasions (following the ingestion of placebo and caffeine), with the results revealing that only the trained swimmers experienced improvements in swimming velocity following caffeine ingestion. Similar findings were observed by Astorino et al. [97], who reported that trained individuals experienced improvements in cycling time-trial performance following caffeine ingestion, while those classified as ‘active’ did not.

Burke [98] argued that trained, as opposed to untrained, individuals might have higher reliability of exercise performance, and, therefore, less day-to-day performance variation. Reliability of performance may be of importance in this line of research, as tests with low reliability may increase the risk of type II errors and are, therefore, not suitable when examining small changes in performance [99]. Hypothetically, in untrained individuals, day-to-day performance variation is greater, potentially preventing the detection of small performance increases following caffeine ingestion. While such ideas are based on a solid rationale [98], studies examining the test–retest reliability of performance tests such as the Yo–Yo test [100], 400-m running [101], power output on the rowing ergometer [102], and 1 RM test [103] in trained and untrained individuals report that both populations exhibit comparable test–retest reliability. These results indicate that other factors are likely responsible for the divergent responses to caffeine ingestion between trained and untrained individuals.

Physiologically, it remains unclear why trained individuals might experience greater performance improvements than untrained subjects following caffeine ingestion. Comparing trained cyclists/triathletes to ‘active’ individuals, Skinner et al. [104] reported that time to peak and peak caffeine concentrations were similar in both groups. Therefore, the availability of caffeine within the blood might not explain the performance differences following caffeine ingestion in trained and untrained individuals noted by Collomp et al. [24] and Astorino et al. [97].

The ergogenic effects of caffeine on exercise performance predominantly appear related to caffeine’s binding to adenosine receptors [105]. Mizuno et al. [106] reported that trained men have greater adenosine A_2a_ receptor densities than untrained subjects; it might be that this increase in adenosine receptor density in trained individuals allows greater binding of caffeine to these receptors, increasing the magnitude of the acute improvements in exercise performance following caffeine ingestion. This idea, however, remains speculative; in contrast to the work by Collomp et al. [24] and Astorino et al. [97], other studies report that caffeine ingestion may produce similar performance benefits in both trained and untrained (or recreationally trained) individuals [107, 109]. Moreover, in some cases, caffeine ingestion enhanced performance in untrained but not in trained individuals [109]; see Table 2 for a summary of these studies.

**Table 2:** Summary of studies exploring the effects of caffeine between trained and untrained individuals

Reference	Sample	Caffeine dose	Performance metric	Main findings
Astorino et al. [97]	8 endurance-trained and 8 ‘active’ young men	5 mg/kg	10-km cycling time trial	Caffeine ingestion reduced the time necessary to complete 10-km of cycling in endurance-trained but not in ‘active’ men
Boyett et al.^a^ [83]	7 endurance-trained and 7 untrained young men	6 mg/kg	Isokinetic knee extension and 3-km cycling time trial	For cycling time trial, the differences in responses to caffeine and placebo ingestion in the morning training sessions were ‘unclear’ between the groups; for the two evening conditions, following caffeine ingestion, untrained individuals ‘likely’ experienced greater reductions in time necessary to complete 3-km of cycling than trained individuals; for isokinetic peak torque, the comparisons were either ‘trivial’ or ‘unclear’
Brooks et al. [109]	7 resistance-trained and 7 untrained young men	5 mg/kg	Weight lifted and force produced in the 1 RM Smith machine squat	Caffeine ingestion improved 1 RM weight lifted in untrained but not in resistance-trained men; no between-group differences were observed for force production
Collomp et al. [24]	7 trained swimmers and 7 untrained swimmers (young men and women)	250 mg	1600-m swimming for the trained swimmers and 400-m for the untrained	Caffeine ingestion improved swimming velocity in trained but not in untrained participants
O’Rourke et al. [108]	15 young well-trained and 15 recreational runners (sex was not specified)	5 mg/kg	5-km running time trial	Caffeine ingestion reduced time necessary to complete 5-km of running in both well-trained and recreational runners
Porterfield et al. [107]	10 endurance-trained and 10 untrained young men	5 mg/kg	Cycling time to exhaustion	Caffeine ingestion did not improve time to exhaustion either in endurance-trained or untrained men

*1* *RM* 1 repetition maximum

^a^Data were analyzed using the magnitude based inferences approach

However, we can potentially gain some insights from animal studies utilizing isolated skeletal muscle, which provide a potentially useful model for the exploration of caffeine’s ergogenic effects as animals tend not to be habitual users of caffeine, and, by bathing the muscle in a caffeine solution, differences in caffeine digestion and pharmacokinetics are largely overcome [110]. A recent study utilizing such a model [111], in which mice either underwent an 8-week exercise intervention or acted as an untrained control, suggested that 70 μM of caffeine (representative of a maximal physiological dose [4]) enhanced muscle power to the same extent in the trained and untrained isolated mice muscle. This suggests that training status does not modify caffeine’s ergogenic effects, at least in terms of direct muscle function. Further work in human subjects, including those with longer (i.e., multiple years) training histories, are required to further explore this hypothesis.

From a different perspective, it could be argued that in highly trained individuals (i.e., elite athletes), there is less ‘potential for improvement’ following caffeine ingestion, as these individuals are, by definition, towards the upper end of human exercise performance capabilities and are approaching absolute physical limits [112, 113]. Given the already high performance capabilities of these individuals, it remains unclear if they can be further enhanced following caffeine ingestion. These aspects remain under-explored given the lack of studies conducted on elite athletes. Due to the limited access to elite athletes and the finite nature of these individuals, even further case studies on this topic would help to expand our current understanding of this topic [114].

In summary, the current evidence on the effects of caffeine among trained and untrained individuals is based on a handful of human studies which reported conflicting findings. This lack of studies opens up an avenue for researchers to conduct future high-quality studies on the topic as this is an area that merits further work.

### Does Caffeine Ergogenicity Vary According to Sex?

A major limitation of the present body of research on caffeine’s ergogenic effects is that the majority of studies utilize male subjects [20, 43]. As an illustration, a recent meta-analysis included nine studies, consisting of 88 pooled participants, of which only three were female [14]. A likely explanation for this difference between sexes is that females represent a slightly more complex cohort to conduct caffeine research on, as the use of oral contraceptives [115] and differences in menstrual cycle stage [116] can alter caffeine metabolization speeds, which in turn may alter the ergogenic effects of caffeine. However, a number of studies demonstrate that caffeine has an ergogenic effect in females, both in terms of resistance [117, 118] and endurance exercise [119, 120]. As such, caffeine is clearly ergogenic for females, but questions remain as to whether there are differences in optimal caffeine strategies between the sexes, especially given the differences in caffeine metabolization speed between males and females.

Several studies compare the effects of caffeine amongst males and females, reporting similar effects in both sexes [121–123], outlined in Table 3. Whilst other studies have recruited both sexes, they generally analyzed males and females together [107, 124]. However, in order to expand our understanding of potential sex differences in response to caffeine ingestion, further work should aim to recruit males and females, and undertake between-group comparisons of the sexes. A similar approach was utilized by Skinner et al. [120], who reported that the magnitude of ergogenic effects following ingestion of 3 mg/kg caffeine 90-min prior to a cycle test was the same (~ 4%) between the sexes. However, there were significant differences in caffeine concentrations post-exercise, with females having a greater amount. This suggests that females do not metabolize caffeine as rapidly as males, hinting that, similar to *CYP1A2* CC genotypes, females might benefit from a longer time between caffeine ingestion and exercise trials [67]; such a hypothesis requires testing.

**Table 3:** Summary of some studies exploring the effects of caffeine between men and women

Reference	Sample	Caffeine dose	Performance metric	Main findings
Butts and Crowell [121]	13 young men and 15 women	300 mg	Cycling time to exhaustion	Caffeine ingestion did not improve time to exhaustion in both sexes
Sabblah et al. [122]	10 young men and 8 women	5 mg/kg	Weight lifted in the squat and bench press 1 RM; repetitions to muscle failure with 40% 1 RM in the bench press	Caffeine ingestion enhanced weight lifted in the 1 RM bench press in men and women; no effects of caffeine were observed for 1 RM squat and 40% 1 RM in the bench press performance to muscle failure in both sexes
Skinner et al. [120]	16 young men and 11 women	3 mg/kg	Cycling time to exhaustion	Caffeine ingestion improved time to exhaustion both in men and women
Suvi et al. [123]	13 young men and 10 women	6 mg/kg	Walks until volitional exhaustion	Caffeine ingestion did not improve time to exhaustion in both sexes

*1* *RM* 1 repetition maximum

### Does Habitual Caffeine Use Alter Its Ergogenic Effects?

Whilst habituation is commonly identified as a factor modifying the acute response to caffeine supplementation [125], research on this topic demonstrates conflicting findings [126]. A recent review [126] explored the influence of habitual caffeine use on the ergogenic effects of an acute caffeine dose. This subject is surprisingly under-studied in human subjects, with the authors finding only four studies [127–130] utilizing a performance task. Of these, two reported a blunting (but not elimination) of caffeine’s acute ergogenic effects with habitual use [128, 129], and two reported no differences in response to acute caffeine ingestion between individuals with different habitual caffeine intakes [127, 130]. These mixed results drove the authors to propose that the difference between habitual and pre-exercise caffeine dose is potentially important; specifically, they hypothesized that high habitual caffeine users perhaps require a pre-exercise caffeine dose in excess of their habitual intake [126]. Further research is required to better understand whether this is indeed the case, especially in light of the recent findings by Lara et al. [131]. Here, participants undertook a double-blind, placebo-controlled, randomized cross-over study, with subjects participating in two 20-day protocols, one with daily caffeine intake amounting to 3 mg/kg, the other with the consumption of placebo capsules. At regular intervals, the subjects undertook a maximally graded time-to-exhaustion cycle ergometer test, along with a 15-s maximal cycle ergometer sprint, with the exercise bout commencing 60 min following capsule ingestion. The authors found that, whilst caffeine remained ergogenic throughout the 20-day period, its ergogenic effects were attenuated. Such a finding suggests that partial habituation may occur. Conversely, Sabol et al. [132] reported that habitual caffeine use had no effect on the ergogenic effects of caffeine in medicine ball throw and vertical jump tests, with these latest conflicting studies further highlighting the need for additional studies in this area. Relevant studies in this area are outlined in Table 4.

**Table 4:** Summary of studies exploring the effects of caffeine ingestion on exercise performance among individuals with different levels of habitual intake

Reference	Sample	Method of assessing habitual caffeine intake	Caffeine dose	Performance metric	Main findings
Beaumont et al. [129]	18 habitually low caffeine users (< 75 mg/day) randomly ingested caffeine or placebo for 28 days	Semi-quantitative food frequency questionnaire (not reported if validated or not)	3 mg/kg for 20 straight days	60-min cycling followed by maximum work completed in 30 min	Habitual caffeine intake attenuated the effects of caffeine on exercise performance
Bell and McLellan et al. [128]	13 caffeine users (≥ 300 mg/day) and 8 non-users (< 50 mg/day)	Questionnaire (not reported if validated or not)	5 mg/kg	Cycling time to exhaustion	The duration and the magnitude of the ergogenic effects of caffeine was greater in habitual non-users
Dodd et al. [127]	8 caffeine users (> 300 mg/day) and 8 non-users (≤ 25 mg/day)	Questionnaire (not reported if validated or not)	3 and 5 mg/kg	Cycling time to exhaustion	No differences in responses to caffeine ingestion between habitual and non-habitual users in time to exhaustion
Evans et al. [203]	6 caffeine users (> 130 mg/day) and 10 non-users (< 40 mg/day)	Questionnaire (not reported if validated or not)	200 mg	10 × 40-m sprints	Caffeine ingestion attenuated sprint performance decrement only in non-habitual users
Lara et al. [131]	11 habitually low caffeine users (< 50 mg/day) randomly ingested caffeine or placebo for 20 days	A food frequency questionnaire (not reported if validated or not)	3 mg/kg for 20 straight days	Cycling time to exhaustion and 15-s Wingate sprints	Habitual caffeine intake attenuated the effects of caffeine on exercise performance
Glaister et al. [27]	21 men with caffeine intake of 88 ± 87 mg/day	Questionnaire (not reported if validated or not)	5 mg/kg	12 × 30-m sprints	No correlation between habitual caffeine intake and improvements in performance following acute caffeine ingestion
Gonçalves et al. [130]	14 low caffeine users (58 mg/day), 12 moderate caffeine users (143 mg/day) and 14 high caffeine users (351 mg/day)	A validated food frequency questionnaire	6 mg/kg	Cycle ergometer time trial	Time necessary to complete the time trial was reduced in all groups following acute caffeine ingestion with no effect of habitual caffeine intake
Jordan et al. [204]	8 caffeine users (> 300 mg/day) and 10 non-users (≤ 50 mg/day)	Questionnaire created by the authors	6 mg/kg	12 × 30-m sprints	Caffeine ingestion improved best sprint time with no effect of habitual caffeine intake
Sabol et al. [132]	6 caffeine users (> 100 mg/day) and 14 caffeine non-users (≤ 100 mg/day)	A validated food frequency questionnaire	2, 4, and 6 mg/kg	Medicine ball throw and vertical jump	Caffeine ingestion improved medicine ball throw distance (only 6 mg/kg) and vertical jump height (all caffeine doses) with no effect of habitual caffeine intake
Tarnopolsky and Cupido [205]	6 caffeine users (> 500 mg/day) and 6 non-users (< 50 mg/day)	4-day food records	6 mg/kg	Ankle dorsiflexors MVC	No effect of habitual caffeine intake and no effect of caffeine ingestion on MVC
Wiles et al. [206]	18 men with different levels of habitual intake (exact values were not specified)	Questionnaire (not reported if validated or not)	3 g of coffee (150–200 mg of caffeine)	1500-m running time trial	Caffeine ingestion reduced time necessary to complete 1500-m running; based on the rank order of habitual caffeine ingestion there was no effect of habitual caffeine intake (however, this study lacked a proper statistical analysis of the differences between individuals with varying amounts of habitual caffeine intake)

*MVC* maximal voluntary contraction

Alongside the issue of caffeine habituation is the issue of caffeine withdrawal as a potential method to enhance caffeine’s ergogenic effects. This becomes an issue if habitual caffeine use does reduce the performance benefits associated with its ingestion, which, as discussed above, is currently unclear. In a recent review [126], the authors identified three studies directly examining the effects of caffeine withdrawal on exercise performance [133–135], with the finding that a pre-exercise caffeine withdrawal period had no impact on caffeine’s ergogenic effects; this, along with the potential downside of caffeine withdrawal, led the authors to conclude that such a withdrawal period was unnecessary.

Furthermore, several methodological aspects need to be highlighted for future studies in this area. One such aspect is the reliability of the exercise test. For example, Bell and McLellan [128] explored the effects of caffeine ingestion between caffeine users and non-users utilizing a time-to-exhaustion test. This may be relevant, given that these tests have been found to have lower reliability than time-trial tests [101]. Therefore, employing a test with a high measurement error can confound study results, possibly leading to erroneous inferences regarding the effects of caffeine among low and high habitual caffeine users. Similarly, the authors of this study (and other studies; e.g., Dodd et al. [127]) did not report if the questionnaire used for assessing habitual caffeine intake was previously validated. Therefore, future studies should ensure that a validated questionnaire for assessing habitual caffeine intake is used. An additional issue in previous studies investigating this topic is that they classify the participants on a broad binary spectrum as caffeine ‘users’ and ‘non-users.’ For example, Dodd et al. [127] classified non-users as those ingesting 25 mg of caffeine per day, while high users were classified as those that ingesting > 300 mg/day; therefore, individuals in the middle range of daily caffeine intake are disregarded. This may be problematic because the middle range likely represents the majority of the population ingesting caffeine; for example, the average consumption of caffeine among the healthy population in the USA is 165 mg/day [136]. To increase their generalizability, future studies should consider using a more comprehensive range of classifying habitual caffeine users, as recently demonstrated by Gonçalves et al. [130]. Finally, given the wide variation in caffeine concentrations both between caffeine sources and within the same caffeine source at different time points [137, 138], the accurate quantification of habitual caffeine use is problematic [139]. A potential solution is the measurement of objective markers, such as urinary caffeine output or plasma caffeine/caffeine metabolite levels [140, 141], although such an approach increases the methodological challenges.

### How Should Caffeine be Utilized Within Repeated Competitive Bouts?

Athletes are often required to undertake numerous competitive bouts within a short time frame; for example, at the 2017 World Athlete Championships, the semi-finals and finals of the 100 m were separated by less than 3 h. Whilst research has established that caffeine is ergogenic, its use around repeated competitive bouts is poorly understood [142]. There are a number of issues requiring consideration here; if the second competitive bout is within the period of time in which plasma caffeine concentrations are maintained, what effect, if any, does a second caffeine dose have? Similarly, because caffeine can increase work rate [143], is it likely to increase fatigue or muscle damage from the initial competitive bout that will carry over to the subsequent bout—and, if so, does this affect performance [144]? How might this affect subsequent sleep (Sect. 2.1)? To what extent does inter-individual variation modify these factors (Sect. 2.2)? It is important that future research replicates how athletes utilize caffeine in the real world, in order to help develop answers to these questions.

There are some initial attempts to answer these questions. For instance, Bell and McLellan [145] reported that ingestion of 5 mg/kg of caffeine prior to morning exercise acutely enhanced performance, and this ergogenic effect was maintained in a second exercise bout performed 6 h later. The maintenance of the ergogenic effect is likely explained by caffeine’s half-life, which is generally 4–6 h [4]. In the same study, re-dosing with an additional 2.5 mg/kg of caffeine did not further improve afternoon performance, suggesting that re-dosing with caffeine prior to the second exercise bout is potentially unnecessary. In contrast, Negaresh and colleagues [146] explored the impact of single (10 mg/kg or 4 mg/kg) or repeated (5 × 2 mg/kg) caffeine doses on wrestling performance, investigated using five performance tests separated by 45–180 min. The repeated dosing approach enhanced performance relative to placebo in the initial four exercise tests, whilst the 10 mg/kg dose only enhanced the first bout, and 4 mg/kg had no ergogenic effect. Given the limited number of studies conducted thus far, further exploration in this area is required.

### Does Caffeine Modify Training Adaptations?

Caffeine has a clear acute ergogenic effect on exercise performance. Given that the effects of caffeine are repeatable across days [147], ultimately, practitioners are also interested in determining whether the use of caffeine, if continued over time, also impacts long-term training adaptations. The scientific attempts to answer this question are unfortunately almost non-existent. One study worth discussing is that of Malek et al. [148]; here, 36 participants were randomized to receive either a placebo (*n *= 18) or a caffeine-containing supplement (three pills equating a total of 201 mg of caffeine; *n* = 18) each day (on training days, 60-min pre-exercise). The training sessions consisted of treadmill running for 45 min and were conducted three times per week, for 8 weeks. Pre- to post-intervention, both groups experienced improvements in peak oxygen consumption (*V*O_2peak_), with no significant between-group differences. These initial results suggest that chronic caffeine use might not augment adaptations to long-term training. However, while interesting, this study also had several limitations. For example, there was no acute component, and therefore, it remains unclear if this supplement was even effective for acute improvements in performance. Secondly, the participants ingested a supplement that contained caffeine, but also over ten other ingredients. Therefore, any potential effect of caffeine could not be isolated.

Future studies are needed to explore this area as there is a logical basis for which caffeine might augment adaptations to training. In resistance exercise, for example, caffeine can acutely increase total volume load [5]. If continued over time, the cumulative effect of these acute performance increases might also impact hallmark resistance-training adaptations, such as skeletal muscle hypertrophy and increased strength, both of which appear to be augmented with increased volume [149, 150]. Furthermore, as caffeine ingestion pre-exercise may delay exercise-induced fatigue, individuals may be able to train for more extended periods with a higher exercise quality, which, subsequently, may enhance long-term training adaptations. Until future long-term studies are conducted, these ideas remain speculative, and future work is evidently needed.

### How Should Caffeine be Consumed?

In the majority of caffeine-based research studies, caffeine is consumed in its anhydrous, powdered form. However, in practice, athletes consume caffeine via a broad array of mediums, including coffee [151], energy drinks [22], bars and gels [152], nasal sprays [153], and chewing gum [154]. In some cases, the caffeine itself is not ingested, but rinsed round the mouth [155], with the proposed mechanism that the caffeine molecules bind to adenosine receptors found there [156].

A summary of the research into many of these alternative methods of caffeine administration was provided in an excellent recent review by Wickham and Spriet [157], to which interested readers are directed. For many of the alternative caffeine administration methods, there is surprisingly scarce research; for example, only two studies have explored the use of caffeinated gels on sporting performance [157]. Whilst early research suggests such alternative methods may be useful, further work is required to determine their efficacy [157], and we should seek to understand the situations in which each provide the optimal benefit. As an example, when administered in the form of caffeinated chewing gum, caffeine appears to be absorbed more rapidly [158], suggesting that it could be of greater use when a quicker effect is required—for example, during the 15-min half-time period in soccer. Similarly, a caffeine mouth-rinse represents a potentially effective method of providing a final pre-competition/exercise trial caffeine dose, as research has shown ergogenic effects immediately following such a mouth rinse [157]. The use of caffeinated gels may be advantageous as caffeine has been shown to enhance glucose absorption, suggesting that, when combined with the carbohydrate found in caffeinated gels, it may enhance performance to a greater extent than a carbohydrate gel alone [159]. Finally, non-ingestion methods of caffeine administration may also be useful for athletes suffering from gastrointestinal distress around exercise, particularly when considering that caffeine can act as a gastric irritant [160]. Enhancing our knowledge in this area, particularly with regard to the determination of an optimal caffeine dose for each method, whether a dose–response relationship exists, if there are any advantages to repeated intakes during an exercise bout, and the optimal timing of each method, therefore holds wide future promise.

An additional way in which caffeine is ingested is via coffee [161]. Whilst coffee, as a method of caffeine ingestion, has been shown to be ergogenic [162], although not unequivocally [163], the question remains as to whether it is as effective as caffeine in isolation at improving performance. Graham [4] concluded that coffee was “probably inferior to caffeine as an ergogenic aid,” and that some of its multiple compounds may reduce the ergogenic effects of caffeine. Hodgson et al. [151] reported that both caffeine and coffee, standardized to deliver a caffeine dose of 5 mg/kg, were similarly efficacious in enhancing performance above placebo in a cycle ergometer test. Two studies [164, 165] compared the use of coffee and caffeine anhydrous for their ergogenic effects on strength and sprint performance, reporting similar ergogenic effects for both modalities. These results suggest a similar ergogenic effect of both caffeine and coffee, provided the caffeine dose is matched.

Practically, the use of coffee as the main caffeine source by athletes prior to competition may be problematic. Caffeine concentrations vary between coffee blends and brands and in the same coffee source over time [137, 138, 166], although such a problem is not unique to coffee [167]. Additionally, coffee tends to be consumed whilst warm, which may cause issues with thermoregulatory control, especially in warm climates [168], and represents a potential logistical issue, as it needs to be transported to the venue in a container that maintains its temperature. However, for non-athletes looking for a pre-exercise caffeine boost, or for athletes in a more relaxed training environment, there appears to be little downside to the use of coffee as a pre-exercise performance-enhancer. Given the ubiquity of coffee consumption in modern society, further research into the ergogenic effects of coffee, when compared to caffeine alone, is warranted, including that of potential side effects such as gastrointestinal distress [162].

### What is the Optimal Dose of Caffeine?

One of the basic questions for those interested in caffeine supplementation is “What dose of caffeine should I use?” Close scrutiny of current evidence highlights we do not have a clear answer to this question, as the large majority of studies examining the effects of caffeine ingestion on exercise performance use a single dose of caffeine. It, therefore, remains unclear if higher doses of caffeine result in greater performance-enhancing effects.

In general, studies conducted thus far utilizing different doses of caffeine do not necessarily support a linear dose–response relationship between caffeine dose and the magnitude of its ergogenic effect. Pasman et al. [169] used doses of 5, 9, and 13 mg/kg, and reported that all doses were equally effective in enhancing cycling performance. Graham and Spriet [170] compared the effects of 3, 6, and 9 mg/kg on performance in a time to exhaustion test, observing that only the two lower doses enhanced performance. Desbrow et al. [171] compared the effects of 3 versus 6 mg/kg and reported that both doses enhanced aerobic endurance, with no between-dose differences.

However, studies utilizing high-intensity, maximal-exertion exercises performance tests report that, in certain instances, higher doses do result in greater improvements in performance. For example, Astorino et al. [172] reported that 5 mg/kg but not 2 mg/kg of caffeine increased peak torque. Pallarés et al. [173] reported that a dose of 9 mg/kg was ergogenic for contraction velocity in high-load resistance exercise (90% of 1 RM), while doses of 3 and 6 mg/kg were not (similar results were observed for 6-s cycling peak power output in the same study). Sabol et al. [132] found that, compared to placebo, caffeine doses of 2, 4 and 6 mg/kg were all effective in enhancing lower-body ballistic exercise performance; however, only a dose of 6 mg/kg enhanced upper-body ballistic exercise. Similarly, Tallis and Yavuz [174] reported that 6 mg/kg of caffeine, as compared to placebo, enhanced isokinetic force production, whilst a lower dose of caffeine (3 mg/kg) did not.

The divergent results for the effects of varying doses of caffeine in different exercise tasks might be explained by the mechanisms of caffeine. When ingested, caffeine elicits many physiological responses in the body, which makes it difficult to isolate the mechanism by which it enhances performance. However, there is evidence that caffeine’s reduction of rating of perceived exertion (RPE) is one of the primary explanatory reasons for its ergogenic effects on aerobic exercise performance [11]. Caffeine binds to adenosine receptors, subsequently reducing RPE during exercise. McLellan and colleagues [105] suggested a threshold plasma caffeine concentration of 15–20 μM (generally attained from a dose of 3 mg/kg) is required to experience an ergogenic effect during aerobic exercise. In this context, it might be that a lower dose of caffeine (e.g., 3 mg/kg) is sufficient for caffeine to bind to the adenosine receptors, reduce RPE, and enhance aerobic performance. Therefore, for this exercise type, higher doses (e.g., 4–6 mg/kg) may not necessarily produce a larger ergogenic effect. Here, the timing of caffeine ingestion needs to be considered. One hour of exercise has been reported to alter the responses of adenosine receptors [175], which may explain why, in some cases, low doses of caffeine, when administered during exercise, may still increase exercise performance [176].

The primary mechanisms by which caffeine increases muscular strength, muscular endurance, and power performance are potentially related to its ability to augment muscle fiber conduction velocity and motor unit recruitment [177]. It might be that increasing the dose of caffeine also increases these properties in a linear dose–response fashion, potentially explaining the findings of Astorino et al. [172] and Pallarés et al. [173]. Indeed, a meta-analysis by Warren and colleagues [16] reported a dose–response relationship between caffeine dose and muscular endurance, supporting this idea. However, many of the studies exploring the effects of caffeine on strength, power, and muscular endurance generally do not measure caffeine plasma concentrations, making it difficult to determine a minimum plasma concentration for the ergogenic effect of caffeine in this exercise type. Finally, we acknowledge that these ideas are merely hypotheses and remain to be explored in future research.

Alternatively, these contrasting findings might be related to the statistical power of the studies. Most of the studies examining the effects of varying doses of caffeine included fewer than 20 participants [19, 144, 169, 171, 178–180], as detailed in Table 5. In a study with 101 participants, Guest et al. [58] reported that doses of 2 and 4 mg/kg were comparably effective for acute increases in 10-km cycling time trial performance. As such, these results are the most robust evidence that greater improvements in performance are not attained by increasing the dose of caffeine.

**Table 5:** Summary of studies exploring the effects of different doses of caffeine on exercise performance

Reference	Sample	Caffeine dose	Performance metric	Main findings
Anderson et al. [119]	8 young female rowers	6 and 9 mg/kg	2000-m rowing time; average power output	↓ in rowing time only with 9 mg/kg; ↔ between caffeine and placebo for average power output
Arazi et al. [207]	10 teenage female karate athletes	2 and 5 mg/kg	Weight lifted in 1 RM leg press; maximum number of repetitions with 60% of 1 RM; vertical jump height; power during ‘Running-based Anaerobic Sprint Test’	↔ between the caffeine doses and placebo in any of the analyzed outcomes
Astorino et al. [172]	15 young active men	2 and 5 mg/kg	Isokinetic knee extension and knee flexion peak torque, average torque, total work, and average power	↑ in peak knee flexion torque only with 5 mg/kg; ↑ in knee extension and knee flexion total work only with 5 mg/kg; ↑ in knee extension and knee flexion average power only with 5 mg/kg
Bruce et al. [208]	8 male rowers	6 and 9 mg/kg	2000-m rowing time	↓ in rowing time with both caffeine doses; ↔ between caffeine and placebo for average power output
Bugyi [209]	25 young untrained men	84, 162, 250 mg	Maximum isotonic hand contractions	↔ between any of the caffeine doses and placebo
Cohen et al. [210]	7 young men and women; the sample comprised competitive road racers	5 and 9 mg/kg	21-km road race time trial	↔ between any of the caffeine doses and placebo
Del Coso et al. [211]	12 young active women and men	1 and 3 mg/kg	Power output in a half-squat and bench press exercises with loads ranging from 10 to 100% 1 RM	↑ in power output during the half-squat only with 3 mg/kg; ↑ in power output during the bench press only with 3 mg/kg (for some loads, 3 mg/kg was more effective than placebo; for others, only the 3 mg/kg vs 1 mg/kg comparison was significant)
Desbrow et al. [212]	9 well-trained young male cyclists	1.5 and 3 mg/kg	Cycling time trial	↔ between any of the caffeine doses and placebo
Desbrow et al. [171]	16 well-trained young male cyclists	3 and 6 mg/kg	1-h cycling time trial	↓ in cycling time with both caffeine doses
Dodd et al. [127]	17 recreationally trained young men	3 and 5 mg/kg	Cycling time to exhaustion	↔ between any of the caffeine doses and placebo
Ellis et al. [213]	15 youth soccer players	1, 2, and 3 mg/kg	20-m sprint time, arrowhead agility change of direction; countermovement jump height, peak power, average power, peak velocity, and peak force; Yo–Yo test distance	↔ between any of the caffeine doses and placebo for 20-m sprint time; ↓ in right change of direction time with all three caffeine doses; ↓ in left change of direction time only with 2 mg/kg of caffeine; ↑ in vertical jump height only with 3 mg/kg; ↑ in peak and mean power as well as peak velocity and force with all three caffeine doses; ↔ between any of the caffeine doses and placebo for Yo–Yo distance
Glaister et al. [178]	17 young male sport science students	2, 4, 6, 8, and 10 mg/kg	Repeated sprint peak power, mean power, and time to peak power	↔ between any of the caffeine doses and placebo
Graham and Spriet [170]	8 well-trained young male distance runners	3, 6, and 9 mg/kg	Running to exhaustion	↑ in total running time only with 3 and 6 mg/kg
Guest et al. [58]	101 male athletes from various sports	2 and 4 mg/kg	10-km cycling time trial	↓ in cycling time with both caffeine doses
Jacobson and Edwards [214]	36 recreationally active young men and women	300 and 600 mg	Isokinetic knee extension and knee flexion peak torque	↔ between any of the caffeine doses and placebo
Jenkins et al. [178]	13 male cyclists	1, 2, and 3 mg/kg	Work performed during 15-min of cycling	↑ in work performed only with 2 mg/kg
Kovacs et al. [215]	15 young well-trained triathletes or cyclists	2.1, 3.2, and 4.5 mg/kg	1 h cycling time trial	↑ in cycling mean work output with 3.2, and 4.5 mg/kg enhanced performance as compared to placebo and 2.1 mg/kg of caffeine; ↑ in cycling mean work output with 2.1 as compared to placebo
McLellan and Bell [216]	13 young recreationally active men and women	3, 5, 6.1, and 7 mg/kg	Running time to exhaustion	↑ in total running time with all caffeine doses
McNaughton [217]	12 male team sports athletes	5 and 10 mg/kg	Running time to exhaustion	↑ in total running time only with 10 mg/kg
Miller et al. [202]	188 young male students	1 and 3 mg/kg	Forearm flexor MVC	↑ in MVC strength only with 3 mg/kg
Pallarés et al. [173]	13 young resistance-trained men	3, 6, and 9 mg/kg	Barbell velocity in the bench press and squat with loads of 25, 50, 75, and 90% of 1 RM; peak power in a 4-s cycling sprint	↑ in barbell velocity at 25% and 50% of 1 RM with all doses in the bench press and squat; ↑ in barbell velocity at 75% of 1 RM with 6 and 9 mg/kg in the bench press; ↑ in barbell velocity at 90% of 1 RM only with 9 mg/kg in the bench press; ↑ in barbell velocity at 75% of 1 RM with all three caffeine doses in the squat; ↑ in barbell velocity at 90% of 1 RM only with 6 and 9 mg/kg in the squat; ↑ in cycling peak power only with 9 mg/kg
Pasman et al. [169]	9 well-trained young cyclists	5, 9, and 13 mg/kg	Cycling time to exhaustion	↑ in total cycling time with all three caffeine doses
Perkins and Williams [218]	14 female young undergraduate students	4, 7, and 10 mg/kg	Cycling time to exhaustion	↔ between any of the caffeine doses and placebo
Sabol et al. [132]	20 young recreationally trained men	2, 4, and 6 mg/kg	Medicine ball throw distance; vertical jump height	↑ in medicine ball throw distance only with 6 mg/kg; ↑ in vertical jump height with all three caffeine doses
Skinner et al. [219]	10 young competitive male rowers	2, 4, and 6 mg/kg	2000-m rowing time; average power output	↔ between any of the caffeine doses and placebo
Stadheim et al. [220]	8 young trained male cross-country skiers	3 and 4.5 mg/kg	Cross-country, double poling ergometer time trial	↑ in total distance covered with both doses of caffeine
Tallis and Yavuz [174]	10 young recreationally active men	3 and 6 mg/kg	Concentric and eccentric knee extension and elbow flexor strength and total work	↔ between the caffeine doses and placebo in the elbow flexors in any of the analyzed outcomes; ↑ in knee extension force only at high angular velocity with 6 mg/kg of caffeine; ↔ between any of the caffeine doses and placebo in average concentric, maximal and average eccentric strength of the knee extensors; ↔ between any of the caffeine doses and placebo in strength of the knee extensors during a repeated contractions protocol
Trevino et al. [180]	13 young recreationally active male	5 and 10 mg/kg	Elbow flexor MVC and rate of torque development	↔ between any of the caffeine doses and placebo
Turley et al. [221]	26 boys	1, 3, and 5 mg/kg	Peak and average power during a 30-s Wingate test; handgrip MVC	↑ in peak power only with 3 mg/kg of caffeine; ↑ in mean power only with 5 mg/kg of caffeine; ↑ in handgrip MVC only with 3 and 5 mg/kg of caffeine

*1* *RM* 1 repetition maximum, *MVC* maximal voluntary contraction, ↑ denotes significant increases, ↔ denotes no significant differences, ↓ denotes significant decreases

Future studies with larger sample sizes are needed to truly determine the optimal caffeine dose. Future studies should also employ performance tests for which it is well-established that caffeine is ergogenic. For instance, Trevino et al. [180] compared the effects of 5 and 10 mg/kg of caffeine on isometric strength. Their results indicated that neither of the doses were effective for acute increases in strength; however, they tested the strength of the elbow flexors, and previous research has demonstrated that caffeine is not always ergogenic for this muscle group [177].

Until more studies using multiple doses of caffeine on exercise performance are conducted, current conclusions can only be that doses in the range from 2 to 9 mg/kg (when administered in anhydrous, powdered form) are required for acute performance-enhancing effects. Still, there remains a possibility that optimal dosages are administration time- [176], task- [173], and individual-dependent [44], especially given the interactions of dose and genotype in the Guest and colleagues [58] study detailed above. Optimal doses may also depend on caffeine source, as there is evidence that very low doses of caffeine administered in caffeinated-chewing gum (e.g., an absolute dose of 100 mg) are also ergogenic [181]. While the question of the optimal caffeine dose for acute performance enhancement is a basic one, we are currently far from being able to answer it, and future studies should aim to explore this area further.

### Does Age Modify Caffeine Ergogenicity?

Whilst we might typically focus on the ergogenic effects of caffeine in younger participants, caffeine’s performance-enhancing effects may hold promise as a method of acutely enhancing exercise capacity, performance, and activities of daily living in older adults. Research has demonstrated that caffeine is effective in enhancing exercise performance in older adults [124, 182]; however, there is the possibility that the magnitude of ergogenic effects varies with age.

Early work by Swift and Tiplady [183] suggested that older adults were more sensitive to caffeine in terms of psychomotor changes, measured by changes in choice reaction time and attentional levels, compared to younger subjects. Utilizing an isolated mouse muscle methodology, Tallis et al. [184] reported that, whilst caffeine was ergogenic across a range of ages, the magnitude of performance improvements tended to be reduced in the muscles of the oldest (50 weeks) and youngest (3 weeks) mice when compared to middle-aged (10- and 30-week-old) mice. To our knowledge, these studies represent the only research to date directly comparing younger and older subjects in terms of caffeine ergogenicity, demonstrating the potential importance of further work in older adults, particularly given the health benefits of enhanced exercise performance in such a population. Additional nuance may need to be explored here, given the potential negative effects of caffeine ingestion on bone mineral density [185, 186] and blood pressure [187, 188], although caffeine and/or coffee consumption does appear to be protective of cognitive function with ageing [189], and tends to be associated with improved all-cause mortality [190].

## Conclusion

Whilst caffeine has a clear, well-established performance-enhancing effect on exercise performance [5–18, 191], there are still many practical aspects regarding its use within sport and exercise that remain poorly understood. The aspects introduced here represent our thoughts on areas that require further exploration in order to better inform and enhance caffeine use strategies in athletes. They are not exhaustive, with other authors having suggested additional aspects. For example, Burke [142] explored the interaction of caffeine with other ingredients, which is an important consideration given that athletes often co-ingest caffeine with other ergogenic aids, such as carbohydrates and taurine in the case of caffeine-containing energy drinks, both of which are themselves capable of exerting ergogenic effects [192, 193]. Additionally, a review by Shabir et al. [194] reported potential expectancy effects of caffeine in 13 out of 17 identified studies, suggesting that if an individual believes they have consumed caffeine, and believes that caffeine is ergogenic, they are likely to experience a performance benefit, even if caffeine has not been consumed. Similarly, correct identification of administration of caffeine or placebo by study participants can modify their performance [195]. As a result, researchers should attempt to control for caffeine expectancy in intervention studies, and also collect data as to the effectiveness of their blinding procedures. Finally, given that the majority of studies on the topic of caffeine and exercise explore its effects only in laboratory conditions, as noted by Burke [98], further research should seek to replicate actual practices in sport, similar to those demonstrated in recent studies across a variety of sports, including volleyball [196], rugby [23, 197], soccer [198, 199], basketball [200], and swimming [201].

Accordingly, it is clear that our understanding of some of the nuances of caffeine and performance remains incomplete. We hope that the aspects identified within this review provide some directions for future research, allowing athletes to better harness the ergogenic effects of caffeine.
